# Genotype–Phenotype Associations in 72 Adults with Suspected *ALPL-*Associated Hypophosphatasia

**DOI:** 10.1007/s00223-020-00771-7

**Published:** 2020-11-15

**Authors:** Nico Maximilian Jandl, Tobias Schmidt, Tim Rolvien, Julian Stürznickel, Konstantin Chrysostomou, Emil von Vopelius, Alexander E. Volk, Thorsten Schinke, Christian Kubisch, Michael Amling, Florian Barvencik

**Affiliations:** 1 Department of Osteology and Biomechanics, University Medical Center Hamburg-Eppendorf, Lottestrasse 59, 22529 Hamburg, Germany; 2 Department of Orthopedics, University Medical Center Hamburg-Eppendorf, Martinistrasse 52, 20246 Hamburg, Germany; 3 Institute of Human Genetics, University Medical Center Hamburg-Eppendorf, Martinistrasse 52, 20246 Hamburg, Germany

**Keywords:** HPP, Alkaline phosphatase, TNSALP, ALP, Pyridoxal 5′-phosphate, PLP

## Abstract

Hypophosphatasia (HPP) is a rare inborn error of metabolism due to a decreased activity of tissue nonspecific alkaline phosphatase (TNSALP). As the onset and severity of HPP are heterogenous, it can be challenging to determine the pathogenicity of detected rare *ALPL* variants in symptomatic patients. We aimed to characterize patients with rare *ALPL* variants to propose which patients can be diagnosed with adult HPP. We included 72 patients with (1) clinical symptoms of adult HPP or positive family history and (2) low TNSALP activity and/or high pyridoxal 5′-phosphate (PLP) levels, who underwent *ALPL* gene sequencing. The patients were analyzed and divided into three groups depending on *ALPL* variant pathogenicity according to the classification of the American College of Medical Genetics and Genomics (ACMG). Reported pathogenic (*n* = 34 patients), rare (*n* = 17) and common (*n* = 21) *ALPL* variants only were found. Muscular complaints were the most frequent symptoms (> 80%), followed by bone affection (> 50%). Tooth involvement was significantly more common in patients with pathogenic or rare *ALPL* variants. Seven rare variants could be classified as likely pathogenic (ACMG class 4) of which five have not yet been described. Inconclusive genetic findings and less specific symptoms make diagnosis difficult in cases where adult HPP is not obvious. As not every pathogenic or rare *ALPL* variant leads to a manifestation of HPP, only patients with bone complications and at least one additional complication concerning teeth, muscle, central nervous and mental system, repeated low TNSALP activity and high PLP levels should be diagnosed as adult HPP if rare *ALPL* gene variants of ACMG class 4 or higher support the diagnosis.

**Electronic supplementary material:**

The online version of this article (10.1007/s00223-020-00771-7) contains supplementary material, which is available to authorized users.

## Introduction

Hypophosphatasia (HPP) is a rare inborn error of metabolism caused by a dysfunction of the tissue nonspecific alkaline phosphatase isoenzyme (TNSALP), which is encoded by *ALPL* [1]. Currently more than 400 different mutations in the *ALPL* gene are known to be responsible for HPP [2]. The clinical spectrum of complications is variable depending on the age of onset, type of mutation and mode of inheritance. At one extreme of the spectrum is the perinatal form, which was usually fatal before the introduction of specific enzyme replacement therapy, as a lack of or low skeletal mineralization led to chest deformities and respiratory failure. At the other end, there is the adult form with typical symptoms such as recurrent stress fractures with associated healing disorders or osteomalacia. The mildest form of HPP is adult odonto HPP, which manifests itself exclusively at the teeth. A total of six clinical subtypes can be differentiated (perinatal, benign prenatal, infantile, childhood, adult, odonto HPP) with continuous overlap [3, 4]. Severe forms of HPP have a reported prevalence between 1/100,000 and 1/300,000, whereas milder forms of HPP are more frequent [5].

In general, typical symptoms of HPP are bone and tooth mineralization disorders, but also less specific symptoms such as musculoskeletal pain, muscular weakness, calcifications of tendons, joints or kidneys and migraine [6, 7]. Low serum TNSALP activity is a hallmark of *ALPL* gene mutations [8] and patients with severe HPP show reduced age- and gender-specific TNSALP activity and accumulation of its substrates, e.g., pyridoxal 5′-phosphate (PLP) [9], which is the hepatic converted form of dietary vitamin B6. Circulating TNSALP activity may be influenced by other factors, which may explain why PLP is reported to correlate better with disease severity in adult HPP [6, 10]. Bone biopsies previously revealed osteomalacia (i.e., osteoid accumulation) depending on disease severity, onset and progression as the cause of skeletal complications [11–13].

Based on the typical clinical symptoms and laboratory constellations, genetic testing is important to support the diagnosis and to exclude differential diagnoses of HPP, e.g., osteogenesis imperfecta or cleidocranial dysplasia [1, 3].

Genetic testing should also be offered to assess the likelihood of disease recurrence for future pregnancies and to achieve a familial analysis for the documentation of the inheritance pattern. If the medical record and a low TNSALP activity of additional family members give clues for carrying *ALPL* mutations, genetic testing should be pursued. Prenatal screening can be offered to families with *ALPL* mutations in case of a high probability for the occurrence of severe HPP, i.e., the presence of at least two affected alleles [1, 3]. The entire clinical presentation consisting of symptoms, typical complications and laboratory changes are crucial for the classification of an *ALPL* variant, as not all detected *ALPL* variants lead to the occurrence of HPP [3, 8, 14, 15]. In Europe, the estimated carrier frequency is around 1/300, but only 4 percent of heterozygote carriers of *ALPL* mutations are estimated to express mild HPP [3]. The judgement of the respective relevance of rare *ALPL* variants for the diagnosis of HPP is currently a challenge for clinicians, which is further broken down in this article. We analyzed a large adult patient cohort with reported pathogenic, (novel) rare and reported common *ALPL* variants only. This work aimed to show clinical and laboratory similarities and differences between patients with reported pathogenic, rare, or common *ALPL* variants only and to propose which patients with rare variants can be diagnosed with adult HPP.

## Materials and methods

### Patients

**Fig. 1:**
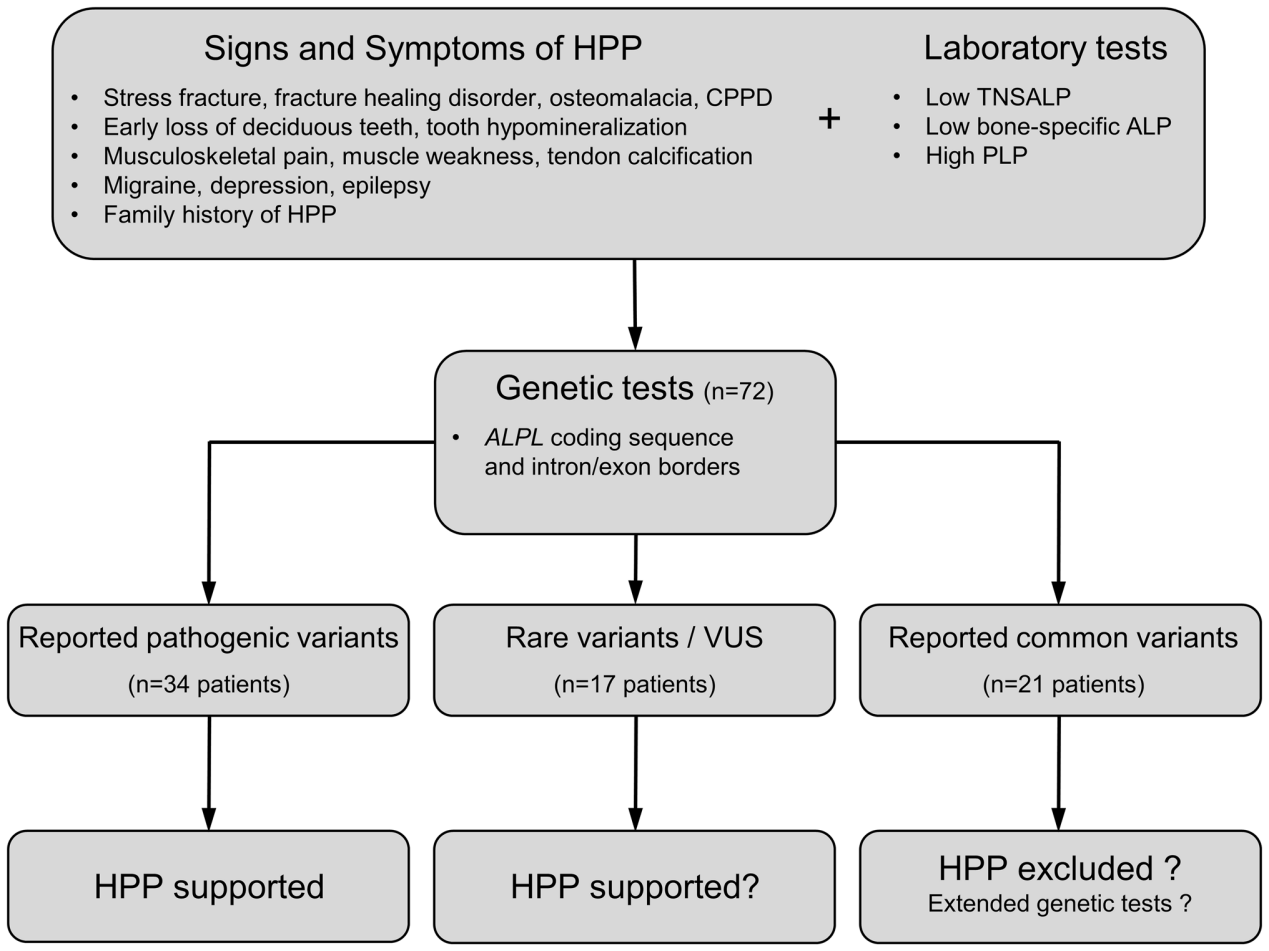
Flowchart with the clinical workflow for patients with signs and symptoms of HPP or a family history of HPP. After detection of laboratory abnormalities with low tissue nonspecific alkaline phosphatase (TNSALP) or bone-specific alkaline phosphatase (BAP) activity and high pyridoxal 5′-phosphate (PLP), *ALPL* gene sequencing is routinely performed. Three groups of *ALPL* variants may be distinguished: (1) Reported variants, that are listed in the *ALPL* gene mutations database by the SESEP laboratory [2] and classified as pathogenic variants, (2) rare variants as well as variants of uncertain significance (VUS), and (3) reported common variants representing *ALPL* polymorphisms without pathogenicity. If a reported pathogenic variant is found, the diagnosis of HPP is supported by the genetic test result. In the presence of a rare variant, the diagnosis must be strictly related to the clinical presentation of the patient, and diagnosing HPP can be challenging in mild forms of adult HPP. The detection of a rare variant with unknown pathogenicity does not support the physician in diagnosing mild adult HPP. Furthermore, it is even more difficult for the physician to assess the presence of HPP if only reported common variants are found in a symptomatic patient. If necessary, more extensive genetic investigations (quantitative multiplex PCR of short fluorescent fragments, exome or genome analysis) could provide additional diagnostic certainty in the future, as this allows the detection of large deletions in the *ALPL* gene and the exclusion of differential diagnoses

We included all adult patients aged > 18 years (*n* = 72) that presented between 2017 and 2019 in our specialized outpatient clinic for bone diseases who fulfilled the following (1) clinical/anamnestic and (2) biochemical inclusion criteria (Fig. 1):

- typical clinical HPP complications, such as recurrent stress fractures, pseudofractures, osteomalacia, fracture healing disorders or dental abnormalities (including early loss of deciduous teeth before the age of 5 years with intact roots, extraction of several permanent teeth before the age of 50 years due to severe caries, narrow jaw or tooth loosening, malocclusion, severe periodontitis, atypical tooth morphology or visible dental hypomineralization) and/or
- less specific symptoms of adult HPP (including pyrophosphate arthropathy/pseudogout/chondrocalcinosis, low bone mineral density with T-Score ≤ −2.5, musculoskeletal pain, weakness, calcifications, neurological/psychiatric symptoms such as frequent cephalgia, migraine or depression) and/or
- a positive family history of HPP,
  in each case (1a–c) together with
- repeated TNSALP and/or bone-specific alkaline phosphatase (BAP) below the reference range or around the limit value to take into account 2 within-lab standard deviations (± 6 U/l) of the test (Supplementary Table, further details see below) and/or
- repeated PLP measurements above the reference range.

The included patients came from 65 families, 13 patients were first-degree relatives, who in turn came from 6 different families. A genetic examination of the *ALPL* gene (further details see below) was performed on all patients as part of the routine diagnostic workup of suspected HPP in our clinic leading to three patient subgroups: (1) patients with reported pathogenic compound-heterozygous (*n* = 3) or heterozygous *ALPL* variants (*n* = 31), (2) patients with rare *ALPL* variants (*n* = 17) defined by an allele frequency < 0.01 according to the Genome Aggregation Database (gnomAD) if available and (3) patients with reported common *ALPL* variants only (*n* = 21). Besides, common variants were also found in some patients with pathogenic or rare variants, but not studied more specifically for these two groups.

Pathogenic and common variants were classified as ‘reported’ according to listings in the *ALPL* gene mutation database by the University of Versailles-Saint Quentin (initiated by the SESEP laboratory) [2]. Among the pathogenic mutations listed in the database are those that occur alone, i.e., in a heterozygous form, and those that were classified as pathogenic together with a second mutation, i.e., as part of a biallelic mutation.

Rare variants were partly described in previous publications, different databases including the Human Genome Mutation Database (HGMD), Single Nucleotide Polymorphism Database (dbSNP) by the National Center for Biotechnology Information (NCBI) and SESEP [2].

Differential diagnoses of low TNSALP activity including osteogenesis imperfecta, multiple myeloma, renal dystrophy, zinc or magnesium deficiency, hypothyroidism, antiresorptive treatment or steroid therapy [1, 4] were excluded by further laboratory tests and clinical examination. Vitamin B6 supplementation as a potential confounder of the serum PLP levels was excluded.

All procedures incorporated in this study were performed in accordance with the Declaration of Helsinki and approved by the local ethics committee (WF-055/19).

### Diagnostics

Bone metabolism parameters including serum TNSALP, BAP, PLP, 25-OH-D3, parathyroid hormone, osteocalcin, calcium, phosphate, and urinary levels of deoxypyridinoline were determined in all patients. TNSALP and BAP reference values were age- and gender-specific (Supplementary Table). Serum TNSALP activity was measured by a Dimension Vista 1500 (Siemens, Erlangen, Germany) using a kinetic rate method as previously described [6]. The reference range used for TNSALP activity was based on a former publication [16, 17], as the manufacturer does not provide gender-specific reference values. BAP was calculated using the LIAISON® BAP OSTASE® assay (DiaSorin, Saluggia, Italy), a one-step delayed addition sandwich chemiluminescence immunoassay (CLIA). The reference range for BAP comes from the manufacturer’s information of the test. It was determined using a sample of 360 healthy individuals.

PLP was determined at the Lademannbogen Laboratory (Hamburg, Germany) using high-performance liquid chromatography with an upper reference threshold of 18.5 µg/l.

Bone mineral density (BMD) at the total proximal femur and the lumbar spine (L1-L4) was measured by DXA (Lunar iDXA, GE Healthcare, Madison, WI, USA) in all patients and corresponding T- and Z-scores were calculated by the software supplied by the manufacturer.

Muscle strength was determined by digital hand dynamometry (Leonardo Mechanograph GF®, Novotec Medical GmbH, Pforzheim, Germany) and the corresponding manufacturer software. The highest measurement out of three tests for the left and right hand, respectively, was considered and the standard deviation (SD) compared to age- and gender-specific reference values was provided [18]. Grip force in kilogram was classified as reduced if there was a decrease of more than two standard deviations for both hands.

### *ALPL* Gene Analysis and In Silico Prediction Tools

Genetic testing was performed at the Institute of Human Genetics at the University Medical Center Hamburg-Eppendorf. DNA was isolated from the EDTA blood sample and an *ALPL* sequence analysis was performed. PCR was used to amplify exons 1–12 (5′-UTR and entire coding region) including flanking sequences of the DNA. To detect molecular changes, the PCR fragments were sequenced directly after purification. The products were analyzed using an automatic sequencer (Applied Biosystems ABI3500). Sequence evaluation was performed with the Sequence Pilot Software (JSI medical Systems, Ettenheim, Germany). The reference sequence was NM_000478.4. The nomenclature of all variants was based on the recommendations of the Human Genome Variation Society. The variants were classified according to the standardized five-stage classification system of the American College of Medical Genetics and Genomics (ACMG) [19]. This classification system for the interpretation of variants divides into benign (class 1) and likely benign variants (class 2), variants of uncertain significance (VUS, class 3) as well as likely pathogenic (class 4) and pathogenic variants (class 5).

Using Combined Annotation-Dependent Depletion (CADD) [20] and Rare Exome Variant Ensemble Learner (REVEL) [21] as prediction tools, we additionally aimed to evaluate the pathogenicity of the rare variants in our cohort. A scaled CADD score greater than 10 indicates that the variant is predicted to belong to the 10% most harmful substitutions in the human genome, a scaled CADD score greater than 20 indicates that the variant is predicted to belong to the 1% most harmful ones. The REVEL score for a variant can range from zero to one. A REVEL score above 0.5 is found in 75.4% of pathogenic mutations but only in 10.9% of neutral variants.

### Statistical Analysis

If not stated otherwise, we report the median and the interquartile range (IQR) of the data. Normal data distribution was tested with the Kolmogorow–Smirnow test. If parameters were not normally distributed, relationships between laboratory parameters were tested by Spearman’s rank correlation analysis, otherwise Pearson correlation analysis was used. Group differences regarding the frequency of affected organ systems (bone, teeth, muscle, neurological/psychiatric) or fracture frequency were analyzed using Kruskal–Wallis- and Chi^2^-tests. Differences of body mass index, laboratory and DXA values between the groups with pathogenic, rare, or common variants were evaluated using univariate ANOVAs with age, gender and body mass index (BMI) as covariates. In case of significant ANOVAs, post hoc t-tests for normally distributed data and Mann–Whitney-*U*-tests for non-normally distributed data were performed. Statistical differences were regarded as significant for *p* values < 0.05. All statistical analyses were conducted using SPSS 22.0 software (IBM, Armonk, NY, USA) and visualization was carried out with GraphPad Prism 7 (GraphPad Software, La Jolla, CA, USA) and Microsoft Power Point & Excel 365 (Microsoft, Redmond, Washington).

## Results

**Fig. 2:**
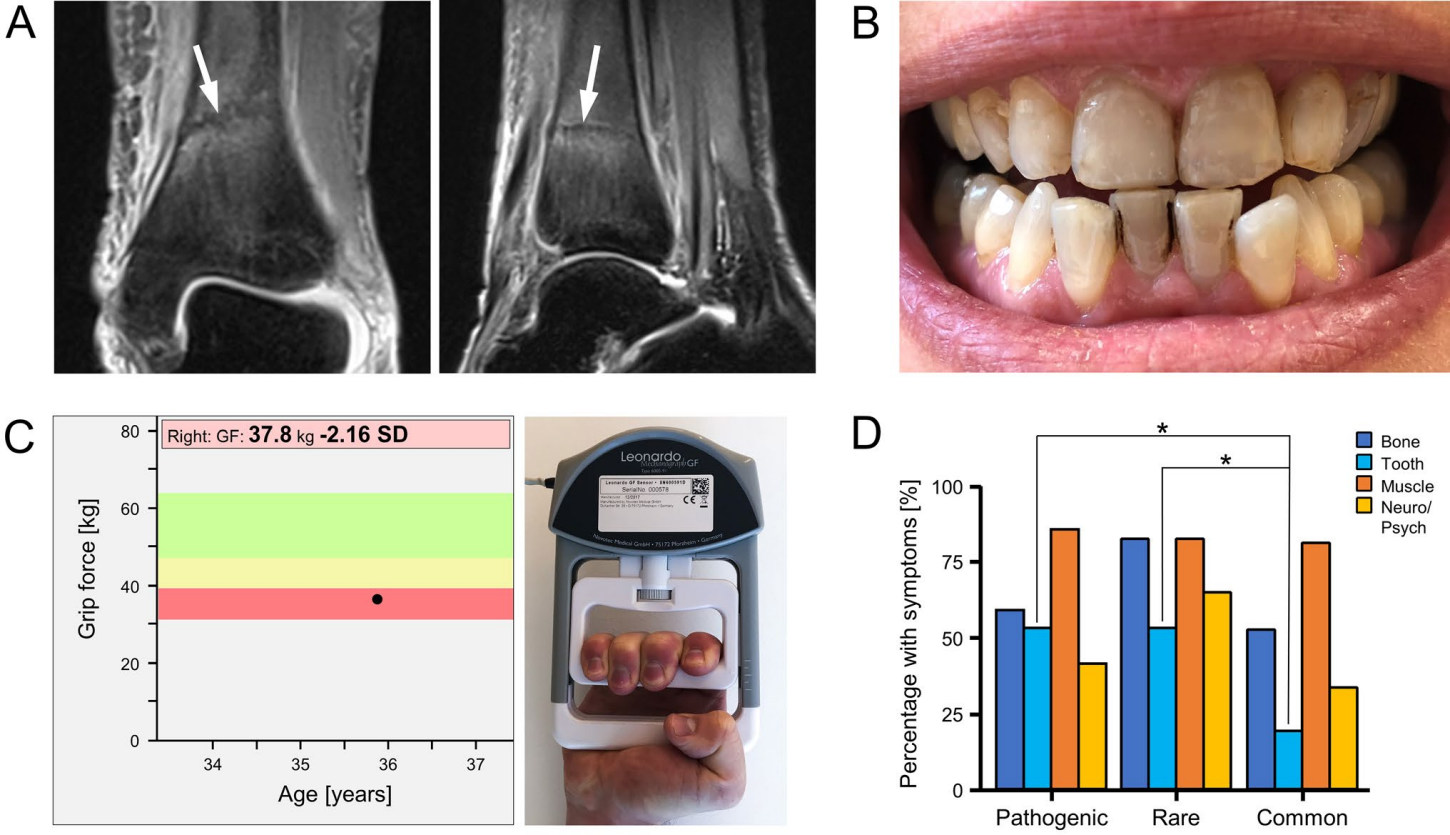
Representative possible clinical complications of adult HPP. **a** MR images (coronal and sagittal, PD-weighted, fat-saturated, turbo spin echo) of a stress fracture of the distal tibia (white arrows), **b** tooth hypomineralization and caries, **c** muscle weakness measured by grip force with digital hand dynamometry. **d** Muscle symptoms were leading in all three patient groups with pathogenic, rare, or common *ALPL* variants. Dental affection was significantly more frequent in patients with pathogenic or rare variants compared to patients with common variants (*p < 0.05)

We analyzed 53 women and 19 men regarding the frequencies of clinical symptoms. Bone involvement manifested as low-traumatic fractures, stress fractures (Fig. 2a), bone marrow edema, fracture healing disorders, T-Score ≤ −2.5, osteonecrosis, and scoliosis. Dental manifestations included early loss of deciduous teeth, tooth extraction (due to severe caries, narrow jaw, or tooth loosening), malocclusion, periodontitis and visible dental hypomineralization (Fig. 2b). Muscle symptoms included muscle pain and weakness, reduced grip force assessed by digital hand dynamometry (Fig. 2c), muscular hypotonia, and tendon calcification. Neurological/psychiatric complaints ranged from frequent cephalgia (more than 15 days per month), migraine, and depression to panic attacks. Muscle symptoms were leading in all three patient groups with pathogenic, rare or common *ALPL* variants only (85% vs. 82% vs. 81%), followed by bone manifestations (59% vs. 82% vs. 52%), dental affection (53% vs. 53% vs. 19%) and neurological/psychiatric symptoms (41% vs. 65% vs. 33%) (Fig. 2d). Dental affection was significantly more frequent in patients with pathogenic variants (*p* = 0.013) or rare (possibly pathogenic) variants (*p* = 0.029) both compared to common variants only.

**Fig. 3:**
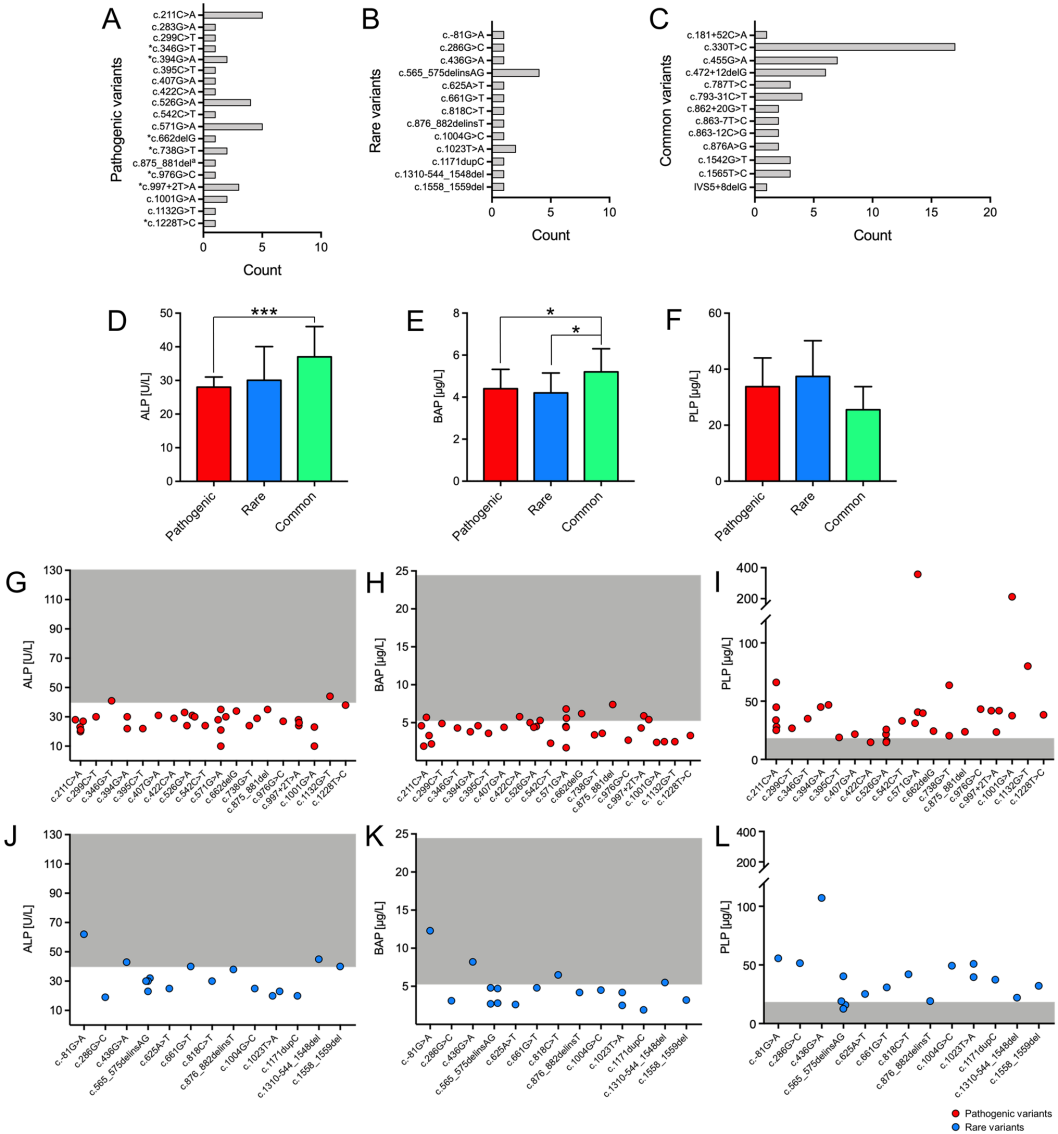
Frequency and laboratory values of patients with reported pathogenic, rare, or common variants in the *ALPL* gene in the overall cohort. **a** All reported pathogenic variants are listed in the hypophosphatasia online database [2] and classified as pathogenic (class 5) according to ACMG criteria. For patients with compound-heterozygous pathogenic *ALPL* variants both variants are listed (c.1001G > A (p.Gly334Asp) + c.283G > A (p.Val95Met)). For patients with one pathogenic and a second VUS (class 3) or likely pathogenic variant, only pathogenic variants are displayed (c.571G > A (p.Glu191Lys) + c.1018C > T (p.His340Tyr), c.542C > T (p.Ser181Leu) + c.818C > T (p.Thr273Met)). ^a^CAGGGGAinsT, *Pathogenic variant, which has so far only been described as pathogenic in combination with a second mutation. **b** Rare variants with allele frequency < 0.01 according to gnomAD (class 3 and 4). **c** Reported common variants classified as benign (class 1) are depicted in the third bar chart. **d**–**f** Median TNSALP, BAP and PLP levels of patients with pathogenic, rare, and common variants. Pathogenic variants were associated with significantly lower TNSALP (***p < 0.001) and BAP (*p = 0.01) compared to patients with common variants. Patients with rare variants showed significantly lower BAP (*p* = 0.043) and borderline significant lower TNSALP (*p* = 0.051) compared to patients with common variants. TNSALP, BAP and PLP did not differ significantly between patients with pathogenic and rare variants. **g**–**i** In 3, 9 and 3 of 34 patients with pathogenic variants, TNSALP and BAP activity as well as PLP levels were in the normal range compared to gender- and age-specific reference values. **j**–**l** In 6, 4 and 2 of 17 patients with rare variants, TNSALP, BAP and PLP levels were in the normal range compared to gender- and age-specific reference values

Analysis of the *ALPL* gene revealed reported heterozygous pathogenic variants in 31 patients and compound-heterozygous pathogenic variants in 3 patients (Fig. 3a). Among all patients with pathogenic *ALPL* variants, 22 patients had a variant that had been reported as being pathogenic in its heterozygous form. In 11 patients the detected heterozygous variant had been described as pathogenic together with a second mutation (marked with asterisks) and in one compound-heterozygous patient both mutations alone have been described as pathogenic in its heterozygous form.

Rare variants were found in 17 patients (Fig. 3b) and heterozygous/homozygous reported common variants only, i.e., frequent, and probably non-pathogenic polymorphisms, in 21 patients (Fig. 3c).

Patients with pathogenic variants showed significantly lower TNSALP (*p* < 0.001) and BAP (*p* = 0.01) compared to patients with common variants (Fig. 3d–f), higher PLP levels failed to reach statistical significance (*p* = 0.072). Also patients with rare variants showed significantly lower BAP (*p* = 0.043) and borderline significant lower TNSALP (*p* = 0.051) compared to patients with common variants only. Interestingly, TNSALP, BAP and PLP did not differ significantly between patients with pathogenic and rare variants. In all three groups, laboratory values indicated a rather low bone formation (reflected by low normal osteocalcin) and normal bone resorption (normal DPD) with a low median TNSALP/BAP activity and consecutively increased median PLP levels. TNSALP and BAP activity were in the lower normal range compared to gender- and age-specific reference values in 3/34 (9%) and 9/34 (26%) patients with pathogenic variants. PLP was in the upper normal range in 3/34 (9%) patients with pathogenic variants (Fig. 3g–i). Rare variants were associated with lower normal TNSALP and BAP activity in 6/17 (35%) and 4/17 (24%) as well as high normal PLP levels in 2/17 (12%) patients (Fig. 3j-l). ANOVAs adjusted for body mass index (BMI) showed significantly lower T- and Z-Scores in the range of osteopenia in patients with rare variants. Table 1 shows bone mineral density and laboratory values of the three groups. 25-OH-D3 was significantly lower in patients with pathogenic variants compared to patients with common variants only. Low-traumatic or atraumatic and stress fracture frequency did not differ between the three groups (35% vs. 35% vs. 39%, *p* = 0.975).

**Table 1:** Bone mineral density and parameters of bone metabolism of patients with pathogenic, rare, and common *ALPL* variants

		Pathogenic (*n* = 34)		Rare (*n* = 17)		Common (*n* = 21)	
		Median	IQR	Median	IQR	Median	IQR
Age		52.0	41.3–59.3	53.0	44.0–62.5	51.0	35.5–68.5
BMI [kg/m^2^]		25.0	22.3–29.7	21.5	20.7–24.7	21.5	19.0–25.6
T-score lumbar spine		− 0.2	− 1.3–1.0	− 1.6	− 2.6–0.0	− 0.9	− 2.0–0.4
Z-score lumbar spine		0.1	− 0.9–1.3	− 0.9	− 1.9–0.6	− 0.3	− 1.2–0.9
T-score left femur		− 0.5	− 1.7–0.0	− 1.9^††^	− 2.9–(− 1.0)	− 1.1^‡^	− 1.8–0.0
Z-score left femur		− 0.3	− 0.7–0.8	− 1.4^††^	− 1.9–(− 0.3)	− 0.5^‡^	− 0.9–0.6
	Reference range						
PLP	< 18.5 µg/l	33.8	23.7–44.0	37.4	20.7–50.2	25.5	18.8–33.8
ALP	35–104/40–129 U/l	28***	23–31	30	23–40	37	31–46
BAP	4.9–26.6/5.2–24.4 µg/l	4.4*	3.2–5.3	4.2	2.8–5.2	5.2^‡^	4.3–6.3
Osteocalcin	5.4–59.1 µg/l	15.4	11.1–18.3	13.3	9.9–17.4	14.5	12.0–16.2
Calcium	2.13–2.63 mmol/l	2.30	2.23–2.33	2.25	2.23–2.35	2.25	2.20–2.32
Phosphate	0.77–1.50 mmol/l	1.13	0.92–1.26	1.08	0.90–1.13	0.98	0.89–1.02
25-OH-D3	30–60 µg/l	26**	21–35	27	23–34	39	25–47
PTH	17–84 µg/l	55	45–71	65	45–76	50	40–62
DPD	3–7 nmol/mmol	5.0	4.0–6.0	6.0	3.5–8.0	5.0	3.0–6.0
Creatinine	0.50–1.00 mg/dl	0.82	0.71–0.96	0.70	0.67–0.88	0.80	0.66–0.90

*BMI* body mass index, *PLP* pyridoxal 5′-phosphate, *ALP* alkaline phosphatase, *BAP* bone-specific alkaline phosphatase, *25-OH-D3* 25-hydroxycholecalciferol, *PTH* parathyroid hormone, *DPD* urinary deoxypyridinoline;

*p < 0.05 (vs. common), **p < 0.01 (vs. common), ***p < 0.001 (vs. common); ††*p* < 0.01 (vs. pathogenic); ‡*p* < 0.05 (vs. rare)

**Fig. 4:**
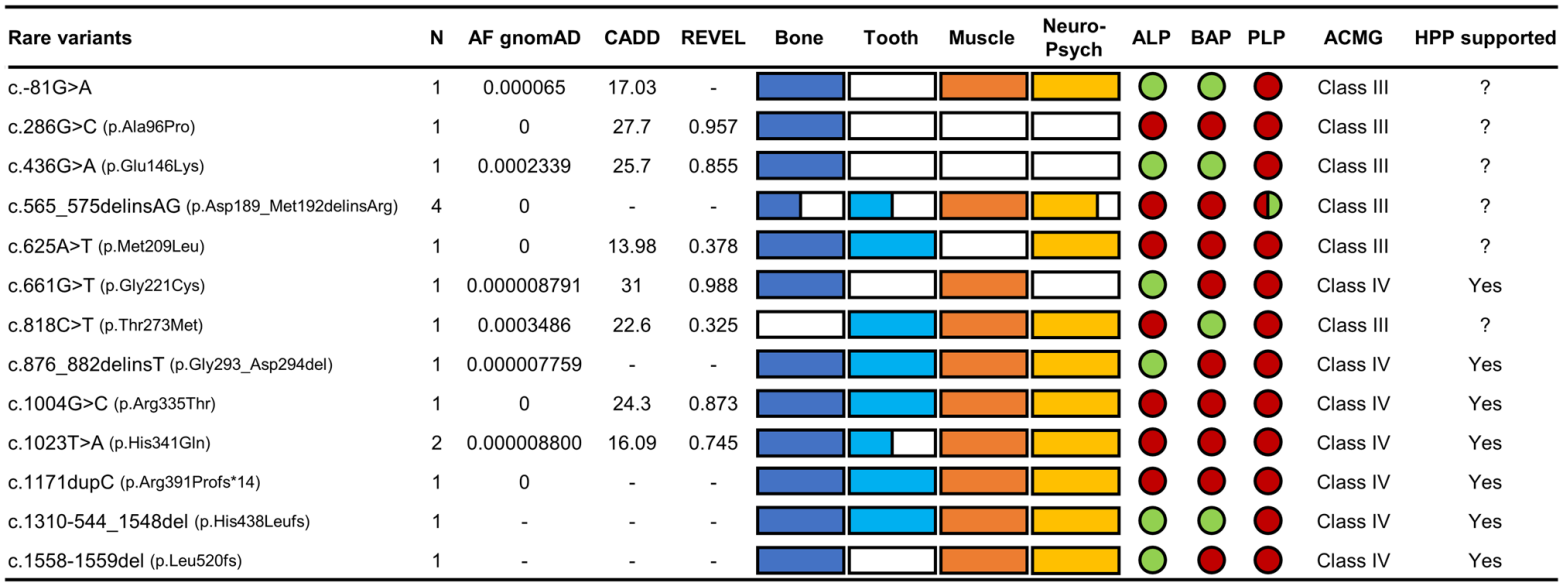
Rare variants with allele frequency (AF) in the European (Non-Finnish) population, in silico prediction results (CADD, REVEL), laboratory values and clinically affected organ systems. Bone (stress fractures, pseudarthrosis, bone marrow edema, low-traumatic fractures, low bone mineral density (T-Score ≤ −2.5), osteonecrosis, scoliosis), tooth (hypomineralization, tooth extraction, early tooth loss, malposition of teeth, periodontitis, caries), muscle (myalgia, tendinopathy, reduced grip force, fatigue) and neurological/psychiatric symptoms (frequent cephalgia, migraine, depression, use of antidepressants). The horizontal boxes indicate the frequency of symptoms for each variant in percentages (full box = 100%, empty box = 0%). The colored circles indicate the proportion of cases for each variant in which alkaline phosphatase (ALP), bone-specific alkaline phosphatase (BAP) and pyridoxal 5′-phosphate (PLP) are pathologic (red: low ALP/BAP, high PLP) or within the lower/upper normal range (green) compared to gender- and age-specific reference values. Adult HPP was diagnosed, if ALP or BAP activity was repeatedly below the reference range or around the lower limit value together with elevated PLP, bone complications and at least one further complication concerning teeth, muscle, central nervous and mental system supported by at least one rare *ALPL* variant (ACMG class 4 or higher)

We further analyzed our subgroup of patients with rare heterozygous *ALPL* gene variants. Table 2 summarizes the genotype–phenotype associations including laboratory values of this subgroup worth scrutinizing. In 16/17 (94%) patients with rare variants, the suspicion of HPP was raised based on typical clinical symptoms. One patient presented due to a family history of HPP (see Table 2, patient 5). TNSALP correlated significantly with BAP (*r* = 0.73; *p* < 0.001), but not with PLP levels (*r* = − 0.16 *p* = 0.552). The two patients with rare variants and low normal PLP levels were both associated with the c.565_575delinsAG variant (Fig. 4). Patients with the same variant c.565_575delinsAG showed variable expressivity with differences in clinical severity and PLP levels. The variant c.1023T > A was found in two sisters both showing similar symptoms and laboratory values (Table 2). Scaled CADD scores of rare variants ranged between 13.98 and 27.7, REVEL scores were between 0.378 and 0.988 indicating different probabilities for pathogenicity.

**Table 2:** Clinical characteristics of patients with rare *ALPL* variants

Pat	Age	Sex	Symptoms	ALP [U/L]	BAP [µg/L]	PLP [µg/L]	Status	Type	References
1	45	M	Low BMD, low-traumatic vertebral fractures, os sacrum stress fracture, clavicula pseudarthrosis, depression, reduced grip force	62	12.3	55.7	het	c.-81G > A	rs528218843
2	64	M	Osteonecrosis	19	3.1	51.5	het	c.286G > C (p.Ala96Pro)	–
3	75	F	Low BMD, low-traumatic wrist fracture	43	8.2	107.2	het	c.436G > A (p.Glu146Lys)	[8, 47], SESEP
4^a^	53	F	Severe caries, migraine, depression, tendinopathy	32	2.8	15.8	het	c.565_575delinsAG (p.Asp189_Met192delinsArg)	SESEP
5^a^	27	F	Cephalgia, reduced grip force	30	4.7	12.6	het		
6^a^	53	F	Low BMD, tooth loss, periodontitis, tooth hypomineralization, narrow jaw, myalgia	30	4.8	19.0	het		
7	48	M	Vertebral bone marrow edema, migraine, reduced grip force	23	2.7	40.3	het		
8	43	F	Tooth extraction, scoliosis, chronic pain, cephalgia	25	2.6	34.7	het	c.625A > T (p.Met209Leu)	[13], SESEP
9	65	F	Low BMD, proximal femur fracture, arthralgia, myalgia	41	4.7	30.1	het	c.661G > T (p.Gly221Cys)	–
10	37	F	Periodontitis, depression, migraine, fatigue, myalgia, reduced grip force	30	6.5	42.1	het	c.818C > T (p.Thr273Met)	rs148405563
11	55	F	Metatarsal stress fractures, tooth loss, depression, migraine, myalgia, fatigue	38	4.2	19.2	het	c.876_882delinsT (p.Gly293_Asp294d)	[33], SESEP
12	49	F	Stress fractures, periodontitis, cephalgia, depression, myalgia, fatigue, reduced grip force	25	4.5	49.4	het	c.1004G > C (p.Arg335Thr)	SESEP
13^b^	57	F	Low BMD, myalgia, tendinosis calcarea, reduced grip force	23	4.2	39.6	het	c.1023T > A (p.His341Gln)	rs1280398041
14^b^	61	F	Low BMD, poor dentition, myalgia, reduced grip force	20	2.5	50.9	het		
15	58	F	Pseudarthrosis, tooth hypomineralization, migraine, myalgia, tendinosis calcarea, reduced grip force	20	1.9	37.4	het	c.1171dupC (p.Arg391Profs*14)	rs751404811
16	35	M	Atraumatic tibia fracture, low BMD, severe caries, dental malposition, depression, panic attacks, reduced grip force	45	5.5	22.1	het	c.1310-544_1548del (p.His438Leufs)	[35], SESEP
17	69	F	Low BMD, bone marrow edema proximal tibia, cephalgia, arthralgia, myalgia, reduced grip force	40	3.2	32.2	het	c.1558_1559del (p.Leu520fs)	rs1553415164

References included literature and the following databases: ALPL Gene Mutation Database (SESEP) and Single Nucleotide Polymorphism Database (dbSNP) by the National Center for Biotechnology Information (NCBI)

*M* male, *F* female, *BMD* bone mineral density, *ALP* alkaline phosphatase (reference range: male 40–129 U/l, female 35–104 U/l), *BAP* bone-specific alkaline phosphatase (reference range: male 5.2–24.4 µg/l, female ≤ 45 years 4.9–26.6 µg/l, female > 45 years 5.5–22.9 µg/l), *PLP* pyridoxal 5′-phosphate (reference range: < 18.5 µg/l); *het* heterozygote)

^a^Members of the same family, two sisters and one daughter

^b^Members of the same family, two sisters

We were able to classify seven rare variants as likely pathogenic (ACMG class 4) and six variants remained of unknown significance (ACMG class 3) based on clinical phenotype, laboratory values, family history, type of mutation, literature and in silico prediction tools (Fig. 4).

As a basis for the diagnosis of adult HPP, we used the measurement of repeatedly reduced TNSALP or BAP activity below the reference range or around the limit value together with elevated PLP, the occurrence of bone complications and at least one additional complication concerning teeth, muscle, central nervous and mental system. If these conditions were met and at least one rare variant with ACMG class 4 or higher within the *ALPL* gene was detected, we diagnosed adult HPP, which applied to 8 of 17 patients.

## Discussion

Whereas patients with perinatal and infantile HPP display severe bone mineralization deficits, rachitic changes and nephrocalcinosis, adult onset HPP shows moderate symptoms like stress fractures with fracture healings disorders, periodontitis, early loss of deciduous teeth and articular calcium pyrophosphate deposition (CPPD) [6, 7]. Less specific symptoms such as muscle pain and weakness are often present [22]. Taken together, the spectrum of complications in adult HPP is very wide [3, 23]. In addition to the measurement of TNSALP and PLP, genetic analysis supports the diagnosis of HPP [3], especially when signs and symptoms are not obvious, as it may be the case in adult HPP. The mode of transmission of the underlying mutation influences the different expressivity of the disease [24]. Severe forms of HPP occur mainly with homozygous or compound-heterozygous mutations, i.e., in an autosomal recessive form of disease [25]. Milder forms of HPP can result from a dominant negative effect (DNE) of heterozygous mutations [26, 27].

In this context, it is difficult to classify rare heterozygous *ALPL* gene variants as disease-causing and thus pathogenic in adult HPP. This study aimed to better characterize rare variants in patients with suspected HPP or a family history of HPP to make a clearer classification, to draw conclusions for potentially further necessary diagnostics and finally to be able to diagnose or rule out HPP. Therefore, laboratory and DXA values as well as clinical symptoms of patients with rare variants were compared with HPP patients with pathogenic variants, and patients with common variants only.

We identified 17 out of 72 patients presenting in our outpatient clinic for bone diseases with clinical symptoms of adult HPP or a family history of the disease and a rare heterozygous *ALPL* variant. Most of these patients showed low serum TNSALP and BAP activity, as well as elevated PLP levels. The median age at the onset of symptoms (51 years) of our study subgroup with rare *ALPL* variants was slightly higher in comparison to other studies on adult HPP [6, 22], but similar to our HPP patients with pathogenic variants within this study. The clinical presentation of our patient subgroup included typical reported HPP complications such as recurrent stress fractures, low-traumatic fractures, pseudarthrosis [22, 28], persistent bone marrow edema [6] and calcific periarthritis [29]. Severe caries, tooth hypomineralization, malposition of teeth, periodontitis and loss of adult dentition were dental manifestations observed, all of which can be symptoms of adult hypophosphatasia [30]. Especially less specific complications were pronounced. Chronic muscle pain as well as muscle weakness measured by grip force, were found as frequent as in the literature [22, 28, 31] and comparable to our adult HPP subgroup in this study. The proportion of patients with frequent cephalgia (53%) or depression (35%) treated with antidepressants was particularly high. A similar observation was made in a previous study [6]. A second study showed a significantly higher prevalence of depression (tenfold) and cephalgia (threefold) among other neurological/psychiatric symptoms in HPP patients compared to the general US population [32]. Whether this can be classified as part of the disease, as a secondary consequence or even independent of HPP exceeds the scope of this study and remains to be further investigated in the future.

According to ACMG criteria we could classify the rare variants c.661G > T (p.Gly221Cys), c.876_882delinsT (p.Gly293_Asp294del), c.1004G > C (p.Arg335Thr), c.1023T > A (p.His341Gln), c.1171dupC (p.Arg391Profs*14), c.1310-544_1548del (p.His438Leufs) and c.1558-1559del (p.Leu520fs) as likely pathogenic (class 4). Taking the clinical presentation, laboratory values and the result of the genetic examination into account, we were able to diagnose adult HPP in the patients with these rare variants. In the following section these particular rare variants will be discussed in more detail and their pathogenicity explained.

The variant c.661G > T (p.Gly221Cys) has not yet been described before. However, known mutations at the same residue are reported to be pathogenic (p.Gly221Arg, p.Gly221Val) in the presence of a second mutation [2]. In silico prediction software CADD predicts that this variant is among the 0.1% most harmful substitutions in the human genome. Our patient showed a T-Score ≤ −2.5, a low-traumatic proximal femur fracture, arthralgia and myalgia accompanied by high PLP, low TNSALP and BAP. Based on two moderate and two supportive criteria [19], we classified the variant as likely pathogenic. Although dental problems did not affect the patient, low-traumatic femur fracture may be a consequence of poor mineralization due to HPP. Additionally, myalgia fits as a less specific symptom, i.e., we diagnosed adult HPP supported by the presence of a likely pathogenic *ALPL* variant. Further evidence for the presence of a severely disturbed enzyme function was provided by Del Angel et al. in a recent study describing a low residual activity of only 4.2% of the wildtype TNSALP by in vitro functional testing of the c.661G > C (p.Gly221Arg) variant [33].

The variant c.876_882delinsT (p.Gly293_Asp294del) was described with a second variant c.962delG causing perinatal HPP [34], whereas only one heterozygous variant was detected in our adult patient. Complications included recurrent metatarsal stress fractures with pseudarthrosis, tooth loss, myalgia, muscle weakness, depression, and migraine, so that we attributed a DNE to this variant based on clinical expression. As the allele frequency according to gnomAD is extremely low and the mother of this patient carried the same variant and presented with CPPD and early tooth loss as previously published [6], we diagnosed HPP and classified this variant as likely pathogenic.

Clinical complications and laboratory values of our patient with the rare variant c.1004G > C (p.Arg335Thr) suggested adult HPP. As this variant is located at or close to the active site of the enzyme [33], has not been reported in controls and there is computational evidence for a deleterious effect, we diagnosed HPP and classified this variant as likely pathogenic. Del Angel et al. described a low residual activity of only 6.0% compared to wildtype activity [33].

The variant c.1171dupC (p.Arg391Profs*14) is a one base pair duplication, predicted to lead to a frame shift and an early stop codon in the mRNA which probably results in a null allele. A base pair deletion (c.1171delC), leading to a frame shift, has already been described in infantile HPP [35]. The patient in our cohort suffered from typical HPP complications and showed a compatible laboratory constellation, which strongly supports the presence of adult HPP.

The variant c.1023T > A (p.His341Arg), located at or close to the active site of the enzyme, was detected in two sisters both presenting with similar musculoskeletal complications and low bone mineral density (T-Score ≤ −2.5). Allele frequency for this variant is low as well as residual TNSALP activity of 5.0%. However, functional testing showed no DNE in vitro [33]. A different pathogenic nucleotide change at this amino acid residue has been determined for the variant c.1022A > G (p.His341Arg) in a case of likely prenatal benign HPP, for which a DNE was found. Therefore, considering the clinical complications, laboratory values and functional in vitro measurements, we diagnosed adult HPP in these two sisters.

The patient with the heterozygous c.1310-544_1548del (p.His438Leufs) variant showed an atraumatic tibial shaft fracture, severe caries, malposition of teeth, depression, and panic attacks. Reduced BMD, low grip force and low normal TNSALP with increased PLP were assessed. However, the initial sequencing of 70 genes for early onset osteoporosis including the *ALPL* gene did not reveal any *ALPL* variants. Due to the strong clinical abnormalities, the genetic diagnosis was extended to genome sequencing and the heterozygous c.1310-544_1548del (p.His438Leufs) variant was finally identified, which probably lead to a loss of the splice-acceptor site and to the deletion of a large part of exon 12. Deletions of exon 12 have been described in the literature together with a second variant as the cause of HPP [36]. It should be emphasized that in this case the TNSALP and BAP were still in the lower normal range after repeated measurements. Nevertheless, the findings in favor of the diagnosis of adult HPP were convincing.

The variant c.1558_1559del (p.Leu520fs) is an unreported frameshift mutation. Our patient showed atraumatic bone marrow edema and low BMD. Additionally, she suffered from arthralgia, tendinopathy and myalgia combined with reduced grip force and frequent cephalgia. Together with reduced TNSALP activity and increased PLP levels, the clinical findings thus support the presence of adult HPP. A similar frameshift mutation c.1559delT has been described for patients with different HPP forms [37], whereas the perinatal severe form was only observed in homozygous c.1559delT patients. The complete absence of TNSALP expression on the cell surface was accounted for perinatal HPP [38]. A DNE could not be ascertained, but the residual TNSALP activity was substantially reduced in vitro compared to wildtype TNSALP activity [33].

However, not all rare variants found were considered to be the causative factor for the patient's complications and remained variants of uncertain significance. It is unclear whether the variant itself can be classified as pathogenic, or whether there are unknown modifier genes or environmental factors that lead to a clinical phenotype in patients carrying these variants. It is known that beside the *ALPL* gene other genes affect TNSALP levels. In a genome wide association study in a large human cohort, other TNSALP level influencing gene loci, namely *GPLD1, JMJD1C*-*REEP3* were identified [39, 40] and the ABO locus is associated with TNSALP levels [41]. Although these gene loci do not cause HPP alone, the combination with *ALPL* variants could trigger a clinical HPP phenotype.

The reasons why six rare variants in this cohort (Fig. 4) are classified as VUS were as follows: In some patients, TNSALP activity was only in the lower reference range (c.-81G > A), PLP changes were inconsistent within a family for the same variant (c.565_575delinsAG), there were few or no typical symptoms or signs of HPP (c.286G > C, c.436G > A, c.625A > T) or skeletal involvement was absent (c.818C > T). Because of these points, a final judgement on the pathogenicity was not possible.

Since not all patients with rare variants and HPP symptoms showed TNSALP/BAP levels below the reference range, the lower limit values for TNSALP and BAP may be too conservative. One reason for this could be that the indicated reference range may not take sufficient account of age and gender variations. In patients with pathogenic *ALPL* variants that were identified by a genetic screening study of unselected patients, more than 40 percent had TNSALP levels above the reference range 40 U/l [42].

Common variants only were found in almost 30% of the included patients who either had evidence of HPP in their family or showed clinical complications comparable with adult HPP. The affected patients showed significantly less tooth involvement, otherwise also typical bone and muscular affection. The absence of tooth involvement seems to be a good indicator to differentiate patients with HPP and pathogenic variants from patients with common variants only. TNSALP and BAP activity in patients with common variants only were reduced compared to the normal range but significantly higher compared to patients with pathogenic or rare *ALPL* variants. It is in line with a recent study comparing hypophosphatasaemic patients with negative genetic *ALPL* testing to patients with pathogenic/likely pathogenic *ALPL* variants [31]. This reflects two aspects: First, our subgroup with common variants includes patients with hypophosphatasaemia who do not have HPP due to few or even no clinical symptoms (inclusion criterion for these patients was HPP in the family). On the other hand, however, it also consists of patients in whom clinically a mild form of adult HPP may be present, but no pathogenic variant could be identified during *ALPL* sequencing. With conventional *ALPL* sequencing, 95% of mutations can be identified, but there is a small diagnostic gap, as the case of the patient with variant c.1310-544_1548del shows. There remains a 5% residual uncertainty for the presence of an undetected variant [43]. In cases of typical clinical complications and low to low normal TNSALP or BAP together with elevated PLP, we suggest a stepwise extension of the genetic testing to quantitative multiplex PCR of short fluorescent fragments [36], exome or genome analysis. In addition, some polymorphisms may have a modifying effect on the occurrence of HPP, for example, low TNSALP activity together with low BMD are described for the common variant c.455G > A [8]. We also found significantly lower T-scores in the subgroup of patients with rare variants, so that an effect on BMD seems probable for some of our detected rare variants as well, compatible with a study examining the association of rare heterozygous variants on BMD [8].

## Limitations

Since patients were referred to us by external clinics and general practitioners for a special consultation for HPP and other bone diseases, a selection bias exists for this cohort. This means that musculoskeletal complications generally occur more frequently in our patient collective than in the normal population. Considering the high prevalence of heterozygous carriers of *ALPL* variants, it is therefore conceivable that some of the associations of complications and genetic findings are random, which is a general limitation of the study. This problem could only be countered by large-scale studies within the normal population.

It should be noted that apart from the rare variants c.565_575delinsAG and c.1023T > A, all other rare variants occurred only once in our cohort, as unfortunately in most cases no larger families could be examined. Therefore, general conclusions regarding dominant negative effects and the penetrance of these variants must be drawn carefully. However, since clear clinical and laboratory criteria of adult HPP as well as ACMG class 4 variants were present in 8 of these patients, we believe that the diagnosis of adult HPP is supported by the genetic result.

A general difficulty lies in the interpretation of heterozygous variants due to a high intrafamily variability. Although the same genotypes in patients with similar clinical findings have been described [3, 24], intrafamily variability was observed not only in heterozygous mutation carriers but also within families with autosomal recessive HPP [44, 45]. This means that a clear distinction between autosomal dominant and autosomal recessive inheritance is not always possible. Furthermore, it remains difficult to predict whether heterozygous variants, which have been classified as pathogenic together with a second mutation, are also pathogenic in its monoallelic form.

As this is not a longitudinal study, it cannot be ruled out that signs and symptoms of the described patients may change in the future and that complications of HPP may occur for the first time or more frequently, which could change the classification of the variants. Especially in adult HPP, the disease often appears not until middle age [6, 27].

Furthermore, a high percentage of residual TNSALP activity for *ALPL* variants compared to the wildtype activity does not exclude the presence of HPP, i.e., results from in vitro studies on enzymatic activity must be interpreted with caution. Other pathogenic mechanisms such as disruption of the TNSALP dimer anchoring, transport, localization and impairment during degradation in the proteasome may be causative for HPP [26, 33, 46]. A certain discrepancy between clinical signs, symptoms and laboratory values was also observed in this study, which makes genetic classification still challenging.

## Conclusions

In this study, we have demonstrated the clinical symptoms, laboratory, and BMD changes in a cohort of 72 adults consisting of patients with HPP and pathogenic *ALPL* variants as well as patients with HPP complications or family history and rare or common *ALPL* variants. We outlined the likelihood of pathogenicity for different rare *ALPL* variants. Since the clinical phenotype, if at all, is only a supporting criterion in the ACMG classification of variants, clinical symptoms and laboratory values should guide the diagnosis of adult HPP. Less specific clinical complications together with decreased TNSALP, increased PLP and inconclusive genetic findings make diagnosis difficult in adult patients. As not every pathogenic or rare *ALPL* variant leads to a manifestation of HPP, we propose that clinical suspicion of the disease characterized by bone complications and at least one additional complication concerning teeth, muscle, central nervous or mental system, and repeated low TNSALP or BAP together with high PLP levels should lead to the diagnosis of HPP if rare *ALPL* gene variants of ACMG class 4 or higher are found. Applying these criteria, we could diagnose adult HPP in more than half of our patients with rare *ALPL* variants.

## Electronic supplementary material

Below is the link to the electronic supplementary material.

Supplementary file1 (DOCX 36 kb)
