# Supplementary material for: Genotype–Phenotype Associations in 72 Adults with Suspected ALPL-Associated Hypophosphatasia
Source: Calcif Tissue Int. 2020 Nov 15;108(3):288–301. doi: 10.1007/s00223-020-00771-7 (PMC7881968; doi:10.1007/s00223-020-00771-7)
Supplement: Supplementary file 1 — Supplementary file1 (DOCX 36 kb) [file 223_2020_771_MOESM1_ESM.docx]

**Supplementary Table: Reference ranges for BAP and ALP**

| **Bone-specific alkaline phosphatase (BAP)** | | |
| --- | --- | --- |
| Age [years] | gender | reference range [µg/l] |
| ≤ 45 | female | 4.9 - 26.6 |
|  | male | 5.2 - 24.4 |
| > 45 | female | 5.5 - 22.9 |
|  | male | 5.2 - 24.4 |
|  |  |  |
| **Alkaline phosphatase (ALP)** | |  |
| Age [years] | gender | reference range [U/l] |
| > 18 | female | 35 - 104 |
|  | male | 40 - 129 |
